# Real-Time Abnormal Object Detection for Video Surveillance in Smart Cities

**DOI:** 10.3390/s22103862

**Published:** 2022-05-19

**Authors:** Palash Yuvraj Ingle, Young-Gab Kim

**Affiliations:** Department of Computer and Information Security, and Convergence Engineering for Intelligent Drone, Sejong University, Seoul 05006, Korea; palash@sju.ac.kr

**Keywords:** deep convolutional network, object detection, gun and knife detection, video surveillance, camera network, computer vision, smart city

## Abstract

With the adaptation of video surveillance in many areas for object detection, monitoring abnormal behavior in several cameras requires constant human tracking for a single camera operative, which is a tedious task. In multiview cameras, accurately detecting different types of guns and knives and classifying them from other video surveillance objects in real-time scenarios is difficult. Most detecting cameras are resource-constrained devices with limited computational capacities. To mitigate this problem, we proposed a resource-constrained lightweight subclass detection method based on a convolutional neural network to classify, locate, and detect different types of guns and knives effectively and efficiently in a real-time environment. In this paper, the detection classifier is a multiclass subclass detection convolutional neural network used to classify object frames into different sub-classes such as abnormal and normal. The achieved mean average precision by the best state-of-the-art framework to detect either a handgun or a knife is 84.21% or 90.20% on a single camera view. After extensive experiments, the best precision obtained by the proposed method for detecting different types of guns and knives was 97.50% on the ImageNet dataset and IMFDB, 90.50% on the open-image dataset, 93% on the Olmos dataset, and 90.7% precision on the multiview cameras. This resource-constrained device has shown a satisfactory result, with a precision score of 85.5% for detection in a multiview camera.

## 1. Introduction

Recently, advancements in video surveillance have driven many new challenges, such as storage, security, and content extraction from videos. Monitoring the videos requires manual human-powered resources, which are error-prone. Many criminal activities, such as robberies, terrorist activities, bomb-blasts, hijackings, and crowd brawl fights, could have been prevented by predicting the threats in advance using video surveillance in real time. Smart city infrastructure is highly dependent on the surveillance system for smooth traffic management and public space monitoring, making the pathways safer and more efficient for every user. Video surveillance, coupled with an object detection mechanism, is a vital tool for analyzing the road networks, intersections, and how people move in the city. In addition, abnormal object detection enables monitoring and tracking of the object of interest more efficiently, so real-time decisions can be made to safeguard or alter the occurring event cautiously. Frequently used objects to commit crimes are guns or knives; globally, there are significant concerns due to increased gun usage for criminal activities, which is validated by statistical reports issued by the united nations office on drugs and crime (UNODC) [1].

One way to prevent such incidents is the early detection of guns and knives at a potential crime scene. A traditional method based on machine learning to detect a gun is using X-rays or scanners (i.e., low-power millimeter-wave radar) [2,3]. Recently, advancements in deep learning, specifically in convolutional neural networks (CNNs), have achieved significant results compared with the traditional machine learning algorithm, such as corner detection and color segmentation techniques employed for object classification, localization, and detection [4,5,6]. Unlike manually selecting features, CNNs focus on extracting rich features automatically [7,8]. When video/image detecting different classes of guns and knives in a real-time situation, various issues can arise, such as more contrast in viewpoints, posture estimation, occlusion, and lighting conditions [9]. These issues lead to difficulties in adequately accomplishing object detection. A large dataset is required to make CNNs more robust for object detection.

Existing studies dealing with these problems are based on different types of deep learning and non-deep learning/classical algorithms for detecting handguns or knives [10,11,12]. Although these studies have produced convincing results for simple cases, their ability to meet compelling circumstances with tricky situations is somewhat limited. Most of the existing studies performed training with a small dataset with fixed constraints for detecting particular objects (e.g., handguns or knives), and most of the techniques were applied on a single camera view. Using an existing algorithm on resource-constrained devices increases the computational cost, leading to poor performance during object detection. Most video surveillance panels comprise multiple cameras; thus, a simultaneous object detection mechanism is essential.

**Figure 1:**
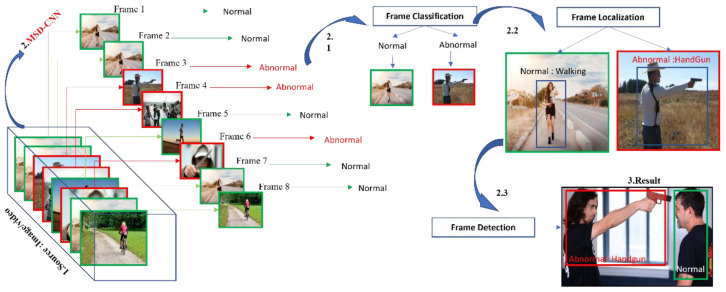
System overview: 1. We first obtain the desired input video or images. 2. On that sequence of frames, the MSD-CNN model is used first. 2.1 Frame classification, 2.2 Frame Localization, and then 2.3 Frame Detection. 3. We can obtain the result.

In this study, to deal with the problems mentioned above, extensive sequences of an experiment were carried out to propose a customized lightweight CNN architecture, called multiclass subclass detection CNN (MSD-CNN). We specifically designed MSD-CNN architecture to detect an abnormal frame in multicamera view using a graphics processing unit (GPU) and dynamic programming for efficiency. The MSD-CNN detects abnormal and normal frames in real time, as shown in Figure 1, and its architecture is depicted in Section 2. To make the MSD-CNN model robust, we significantly increased the training dataset by performing data augmentation [13] on an ImageNet dataset, as described in Section 4.

The proposed model can detect and classify the abnormal and the normal class. There are two prominent (i.e., normal and abnormal) classes for classification, which is referred to as multiclass. The subclass states the significant class corresponding to an appropriate subclass activity, whereas the abnormal class is a frame in which abnormal object activities are present, based on the characteristics of these activities; it gets categorized into two different abnormal subclasses. Similarly, the normal class is a frame where normal object activities are present, further segregated into two normal subclasses. The primary purpose of our model is to detect the different types of subclasses of guns and knives, such as handguns, automatic/semi-automatic rifles, kitchen knives, and army knives, considered to be abnormal frames/objects, and normal frame/object subclasses such as activities and work, which are walking, cycling, office work, and housework.

The main contribution of the study is listed below:

- Our study introduces a new lightweight multiclass-subclass detection CNN model to effectively and efficiently extract and detect abnormal features in a real-time scenario from both multiview and single view cameras;
- To facilitate the learning of the model for real-time detection, we constructed a custom dataset for training;
- We have summarized the insights of different algorithms used to detect handguns and knives and construct a taxonomy;
- We introduce a new evaluation method, Detection Time per Interval (DTpI), to evaluate an object’s emplacement concerning the multiclass evaluation real-time score in a multiview camera;
- The proposed model has achieved a better result than the state-of-the-art framework for detecting abnormal frames in real time.

To the best of our knowledge, this is the first study to consider a multiclass-subclass detection classifier for detecting different types of guns and knives in real time. In Section 2, we discuss the existing detection algorithms and separate them according to their respective characteristics. A detailed description of the proposed architecture of MSD-CNN and the learning algorithm is explained in Section 3. In Section 4, we evaluate the model with existing standard datasets and compare the result with the state-of-the-art algorithms, whilst future enhancement and weaknesses are mentioned in Section 5. Section 6 concludes the study.

## 2. Related Work

Object detection [14] in videos or images can be classified into two broad research areas. The first addresses gun and knife detection using classical/non-deep learning algorithms, whilst the second focuses on improving the object detection accuracy using deep learning algorithms. Basically, non-deep learning/classical algorithms are based on color-based segmentation, coroner detector, and appearance. A disadvantage of using the existing classical algorithm is that it is highly dependent on the quality of frames/images. Frames with occlusion and noise are difficult to interpret; in addition, when foreground and background color segments are matching, interpretation is difficult when using color-based segmentation [15]. Neural networks are basically used in deep learning algorithms. An advantage of using a neural network model is it learns feature extraction automatically through training. A model trained on larger data can detect occluded frames. In a model such as CNN or R-CNN, before training, the data must be labeled, referred to as supervised learning. The algorithms discussed in the following subsections elaborate on the methods and mechanisms used for detection.

### 2.1. Gun and Knife Detection Using a Classical Algorithm

The classical algorithms used for detecting guns and knives are based on various frame segmentation methodologies for detecting and extracting key features from images. In this section, we describe them in detail.

#### 2.1.1. Active Appearance Model (AAM)

The AAM [16] uses a statistical model to match features. Basically, it is used in facial detection. AAM annotates the image and then represents the image in vector form [17]. It uses principle component analysis to normalize the images. T. Rohit et al. [15] used the AAM and trained it on a customized image dataset for detecting knives, thus leading to maximum false positives when detecting. Specifically, the AAM could detect knives with a sharp edge in an image; it requires clear visibility of objects in the image. A disadvantage of AAM is that it poorly detects objects in noisy images.

#### 2.1.2. Harris Corner Detector (HCD)

HCD extracts features from the corners of images. Steps involved in the Harris detection are as follows. First, the image is converted into grayscale. Second, using spatial derivatives, the corners of the image are identified. The detected object’s tensor structure is generated using Harris calculation, and finally, using non-max suppression, the object is determined. A. Glowacz et al. [18] synergistically used the AAM and HCD for detecting guns and knives; for training, they used a customized image dataset and achieved better results than T. Rohit et al. [15]. However, it is time-intensive for processing and thus slow when performing real-time detection.

#### 2.1.3. Color-Based Segmentation (CBS)

CBS uses k-means [19] to find the cluster of the subset. This helps to remove the unwanted colors from the images, and then the HCD algorithm is used to find the object in the image. In their respective studies, T. Rohit et al. [15] and P. Pratihar et al. [16] used both CBS and HCD to detect knives and guns. The model was trained on a customized image dataset. After excluding the unwanted color, HCD was used to detect the appropriate object. The model was only used to detect X-ray images with a maximum number of false positive as the model was trained on a less significant dataset.

### 2.2. Gun and Knife Detection Using Deep Neural Networks (DNNs)

**Figure 2:**
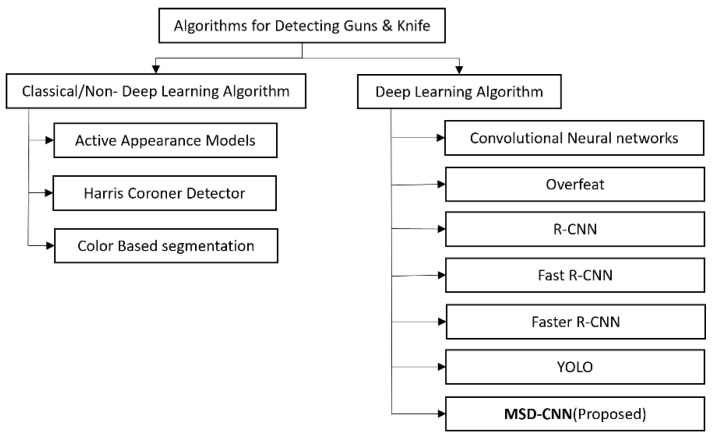
Taxonomy of algorithm for detecting guns and knives.

DNN learning algorithms are built on top of neurons [5]. A DNN comprises several layers, and each of these layers contains neurons; each neuron is defined by input points, hidden points, and output points. These layers are interconnected with each other based on the weights of neurons. The previous neuron output is the input of the next neuron in the layer, which is multiplied by the corresponding weight. All values are summed and added to the defined bias value. The obtained sum becomes an input to the next neuron. The resultant value is passed to an activation function that transforms the parameters and is passed to the next neuron. Likewise, all input values are propagated through the entire neural network. Consequently, the neural network is used to predict the result. The predicted result and the actual result difference is called an error, which is calculated by an error function based on the error value that is generated when updating the weights; this process is repeated until the obtained error does not minimize. A deep learning algorithm is used to detect an object based on the features on which it is trained. The neural network architectures for detecting handguns and knives are CNN, Overfeat, Region-based CNN (R-CNN), Fast R-CNN, and Faster R-CNN. The taxonomy of this algorithm is shown in Figure 2.

#### 2.2.1. Overfeat

Overfeat is based on the sliding window approach of CNN. The sliding window classifier is trained to detect the object at the center and then other parts of the image. Based on this, L. Justin et al. [20] achieved a satisfactory result for detecting a handgun. The model is trained on a standard ImageNet dataset. Notably, it is still significantly slow while detecting the frames in real time.

#### 2.2.2. CNN

Asrith et al. [21] used low-resolution images for training CNNs to detect faces and weapons. Basically, their study focused on face detection and did not contemplate weapon detection. A. Castillo et al. [10] proposed a CNN algorithm to detect a cold metallic weapon in video surveillance. Similarly, work was carried out by F. Gelana et al. [22] to detect a handgun using edge information of the object as a feature, which was based on CNN; the accuracy achieved to classify a frame in CCTV videos was 97.78%.

#### 2.2.3. R-CNN and Faster R-CNN

Dhillon et al. [23] proposed a handgun detector that was trained using an internet movie firearm database (IMFDB) dataset using an R-CNN model whose classification head was constructed on top of VGG16 architecture. They used a support vector machine and ensemble tree classifier for classification, regression, and outlier detection.

In their respective studies, Mikolaj et al. [24,25] and Akcay et al. [26] proposed an X-ray baggage screening system used to classify and detect objects. These studies explored multiple object detection mechanisms such as Faster R-CNN, sliding window CNN, and You Only Look Once (YOLO). This system was proposed to classify the object into six classes such as laptops, guns and their parts, and knives and their parts. However, their proposed model could not detect objects with accuracy if it had high occlusion under different lighting conditions. An action recognition method is used for detecting the anomaly using timed image-based CNN [27].

#### 2.2.4. YOLO

Kanehisa et al. [28] proposed the YOLO algorithm for handgun detection. The dataset used for training was IMFDB. The study to detect a knife with the most pertinent result was obtained from the common object in context (COCO) challenges released in 2017. Object detection in COCO [29] was based on a very large-scale dataset.

Bhatti et al. [30] constructed a customized dataset for training the YOLOv4 model and compared the results with the state-of-the-art methodology. They achieved a satisfactory result by testing their methods on a few videos. Their study mainly focused on detecting a pistol, revolver, wallet, metal detector, and cell phone. In their study, they compared the results with single shot multibox detector (SSD), R-CNN, and a different version of YOLO; some of the classification models have shown promising results in static mode, but in real-time scenarios, the models were slow and less accurate when converging on a resource-constrained device. These studies demonstrated an excellent F1 score on the initial dataset, but the models are not suitable for scenarios with background objects.

**Figure 3:**
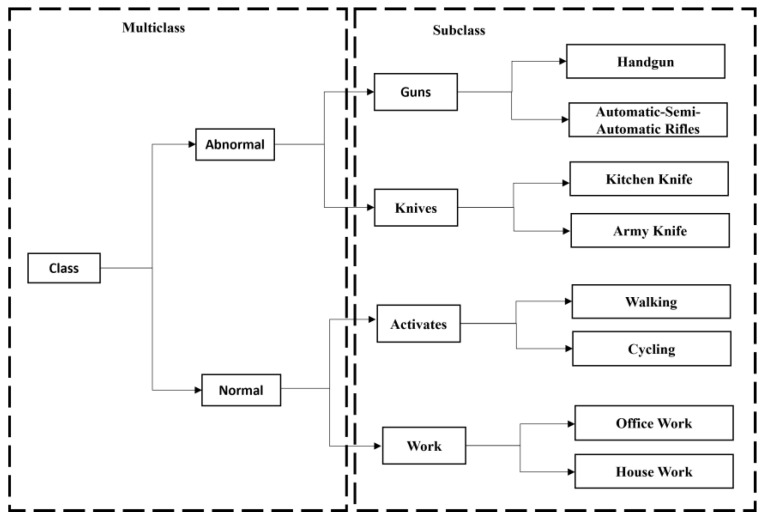
Illustration of multiclass subclass sequential flow. The dashed arrow indicates the abnormal subclass; the dotted arrow indicates the normal subclass. The right-side box indicates the major multiclasses; the left-side box indicates their respective subclasses.

For only detecting handguns, the most prevalent result was obtained using Faster R-CNN [31]; for knives, this was obtained using CNN [32]. As most existing algorithms struggle to detect smaller objects and test them on constrained lightweights devices, the test time significantly increases. As a novelty, this study focused on classifying different subclasses of guns and knives; Figure 3 shows the sequential flow of the different subclass detection.

## 3. MSD-CNN

**Figure 4:**
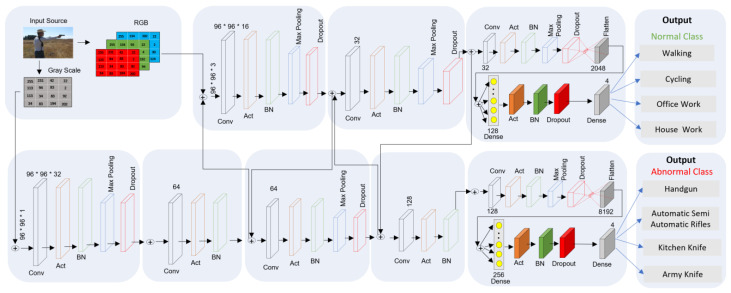
The architecture of the MSD-CNN model. Black, orange, green, blue, and red rectangles indicate the convolutional layer (Conv), activation layer (Act), batch normalization layer (BN), max pooling, and dropout, respectively. Light gray filled rectangles indicate the flattened layer; the dark gray rectangle indicates the dense layer. ⨁ indicates the addition of input features.

We first describe the entire network of MSD-CNN architecture in this section, as shown in Figure 4, followed by the architecture implementation flow. Finally, we describe the detailed MSD-CNN methodology and the proposed learning algorithm.

### 3.1. MSD-CNN Network

MSD-CNN stands for multiclass subclass detection convolutional neural network architecture, as shown in Figure 4. Two fully connected (FC) heads are present in MSD-CNN, and each head has a specific classification task. As there are two separate branches, the branch at the first edge is responsible for classifying the abnormal subclass images such as guns (e.g., handguns and automatic/semi-automatic rifles) and knives (e.g., kitchen knives and army knives). The second edge branch is responsible for classifying the normal subclass images such as activities (e.g., walking and cycling) and work (e.g., office work and housework). The architecture of CNN used here is a simpler version of VGGNet [33,34]. Table 1 shows the notation for presenting the quantities and their respective definitions used in this study.

### 3.2. Architectural Flow

In the proposed architecture, two forks are stacked on top of each other. The right branch/second fork in the network is shallower compared to the left branch/first fork. Predicting the normal class is easier than predicting the abnormal class; thus, the right branch is more superficial. The stacking of multiple layers of CONV (convolutional) and rectified linear unit (RELU) helps the system to learn features with richer characteristics. The architecture accepts input images with dimensions 96 × 96 × 3. In the MSD-CNN network, each branch is responsible for its set of tasks, such as convolution, activation, batch normalization, max pooling, and dropout. There are 32 filters in the CONV layer, with a kernel of 3 × 3 and activation function as a RELU. The RELU activation function is mathematically stated in Equation (1).

(1) y=max  (0,x)

Thus, 25% of dropout is applied in the MSD-CNN network to optimize the hyperconnectivity between the neural network. Dropout is required for disconnecting the nodes randomly from the existing layer to the next layer. Randomly disconnecting nodes helps to reduce overfitting, as a single node is not responsible for predicting a class, edge, or object. In tandem, the filter kernels and pool size are progressively changed to reduce the spatial size and increase the depth.

While training in the first fork, we used grayscale images because we were concerned with very small objects (e.g., handgun, knife) for detection. Also, when training the second fork, we used a colored image and were concerned with larger parameters (e.g., house and building). If we had converted it to grayscale, color information would have been lost, so in order to preserve the tiny object’s information, retaining the color is essential. The stacking of convolutional layers on top of each other helps to increase the depth of the network. Max pooling is used to reduce the volume size. Pooling layers use a 3 × 3 pool size to quickly reduce the spatial dimension from 96 × 96 to 32 × 32. Thus, increasing the filter size of a node from 32 to 64 results in a smaller the spatial dimension of volume; the smaller it becomes, the deeper we go into the network, and the more filters we learn. Mathematically, an image represented in tensor form is

(2) Dim (image)=(nH,wW,nC)

At the *l*th layer, it can be denoted as

- First bullet Input: $a^{\left[\;l-1\;\right]}$, with size $\left(n_{H}^{\left[\;l-1\;\right]},n_{W}^{\left[\;l-1\;\right]},n_{C}^{\left[\;l-1\;\right]}\right)$
- Number of Filters:$\;n_{C}^{\;\left[\;l\;\right]}$, where each $K^{\left(n\right)}$ has dimensions $\left(f^{\left[\;l\;\right]},f^{\left[\;l\;\right]},n_{C}^{\left[\;l-1\;\right]}\right)$
- Activation Function:$\;\varphi^{\left[\;l\;\right]}$
- Output:$\;a^{\left[\;l\;\right]}$, with size $\left(n_{H}^{\left[\;l\;\right]},n_{W}^{\left[\;l\;\right]},n_{C}^{\left[\;l\;\right]}\right)$

So, we have

$$\forall n\in \left[1,2\;\ldots .,n_{c}^{\left[l\right]}\right]\;conv\;\left(a^{\left[\;l-1\;\right]},K^{\left(n\right)}\right)=$$

(3) $$\varphi^{\left[l\right]}\left(\sum_{i=1}^{n_{H}^{\left[l-1\right]}},\sum_{j=1}^{n_{W}^{\left[l-1\right]}},\sum_{K=1}^{n_{C}^{\left[l-1\right]}}K_{j,j,k}^{\left(n\right)}\;a_{x+i-1,y+j-1,k}^{\left[l-1\right]}+b_{n}^{\left[l\right]}\right)$$

(4) $$\;\dim\;\left(conv\;\left(a^{\left[l-1\right]},\;K^{\left(n\right)}\right)\right)=\left(n_{H}^{\left[l\right]},n_{W}^{\left[l\right]}\right)$$

Thus,

(5) $$\left[\varphi^{\left[l\right]}\left(conv\left(a^{\left[l-1\right]},K^{\left(1\right)}\right)\right),\varphi^{\left[l\right]}\left(conv\left(a^{\left[l-1\right]},K^{\left(2\right)}\right)\right),\ldots ,\varphi^{\left[l\right]}\left(conv\left(a^{\left[l-1\right]},K^{n_{C}{}^{\left(l\right)}}\right)\right)\right]$$

(6) $$\dim\left(a^{\left[l\right]}=\left(n_{H}^{\left[l\right]},n_{W}^{\left[l\right]},n_{C}^{\left[l\right]}\right)\right)\;$$

At the *l*th layer, the learned parameters are

- Filters with $(f^{\left[\;l\;\right]},f^{\left[\;l\;\right]},n_{C}^{\left[\;l-1\;\right]})\times n_{C}^{\left[\;l\;\right]}$ parameters
- Bias with (1 × 1 × 1) × $\;n_{C}^{\left[\;l\;\right]}$ parameters

The pooling layer is responsible for downsampling the features of input without affecting the channels; basically, pooling layers have no parameter to learn, FC layers are a finite number of neurons that accept input as a vector and return output as a vector:

(7) $$Y_{j}^{\left[i\right]}=\sum_{l=1}^{n_{i-1}}W_{j,l}^{\left[i\right]}\;a_{l}^{\left[l-1\right]}+b_{j}^{\left[i\right]}\to a_{j}^{\left[i\right]}=\varphi^{\left[i\right]}\left(Y_{j}^{\left[i\right]}\right)$$

The input $a^{\left[\;l-1\;\right]}$ is a result of convolution or a pooling layer with a dimension of $\left(n_{H}^{\left[\;l-1\;\right]},n_{W}^{\left[\;l-1\;\right]},n_{C}^{\left[\;l-1\;\right]}\right)$. To pass the value to the FC layer, we flatten the tensor to 1D dimension:

$$\left(n_{H}^{\left[\;l-1\;\right]},n_{W}^{\left[\;l-1\;\right]},n_{C}^{\left[\;l-1\;\right]},1\right)$$

Thus,

$$n_{i-1}=\left(n_{H}^{\left[\;l-1\;\right]}\;x\;n_{W}^{\left[\;l-1\;\right]}\;x\;n_{C}^{\left[\;l-1\;\right]}\right)$$

Resultant parameters that are learned are

- Weights $W_{j,l}$ with $n_{\;l-1\;}\times n_{\;l}$ parameters
- Bias with $n_{\;l}$ parameters

**Figure 5:**
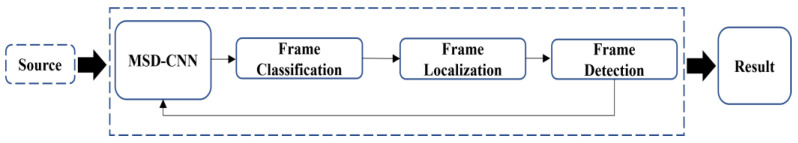
The sequence of the flow of MSD-CNN methodology.

The MSD-CNN methodology, as shown in Figure 5, is comprised of frame classification, localization, and detection. The following component works together in a sequence to detect the desired object. Frame detection is basically used to accurately find the location and dimension of an object in an image, which is essential for frame classification [35].

The position and scale of an object are determined by frame localization [36]. After defining the architecture, the learning algorithm is conducted in the following steps: In the neural network learning algorithm, it is basically a stepwise calculation of each defined layer’s parameters’ weights. The goal is to achieve the best parameters for prediction. The loss function denoted by ***J*** defines the difference between the actual value and the predicate value on the entire training set. To minimize the value of *J*, first, in forward propagation, the data is iterated through the entire network in a sequence of batches, so that the loss function (***ℒ***) for each batch is calculated, where *m* is the size of the training set and θ is the model parameter; consequently, it defines the sum of the errors committed at the predicated outcome of each batch. Second, backpropagation is used for calculating the gradients of the cost function, so that the parameters can be updated using the descent algorithm.

(8) $$J\left(\theta \right)=\frac{1}{m}\;\sum_{i=1}^{m}L(y\;hat_{i}^{\theta },y_{i})$$

Algorithm 1 defines object detection in multiview cameras using GPU: For detecting an abnormal object in multiview cameras in real time, we use the concept of threading and GPU for computation. MSD-CNN is a considerably lightweight network; it is possible to create multiple instances of the MSD-CNN model, thus simultaneously applying the model individually on each video sequence using dynamic programming. Thus, we are able to detect the abnormal object in multiple cameras simultaneously without increasing the computation overhead. The video sequence is transferred from the main memory to global memory to implement the threading and improve the optimization of computational resources. Each instance is responsible for detecting, classifying, and localizing the object in the desired frame for a particular video sequence—evaluating the methods discussed in the experimental Section 4.
**Algorithm 1.** Detection in multiview cameras using GPU.1 Transfer Video Data:from Cameras to Main Memory 2 Transfer Video Data:from Main Memory to Global Memory 3 Fetch video sequence from global memory 4 Threads (videos) 5  Detected_frame_video_sequence_1=      MSD-CNN applied on video_1    /* object classification and localization*/ 6  Detected_frame_video_sequence_n=     MSD-CNN applied on video_n    /* object classification and localization*/

## 4. Evaluation

To evaluate the proposed MSD-CNN, we used three different standardized datasets: ImageNet, Open Image dataset V4 [11], and Olmos dataset [36].

**Figure 6:**
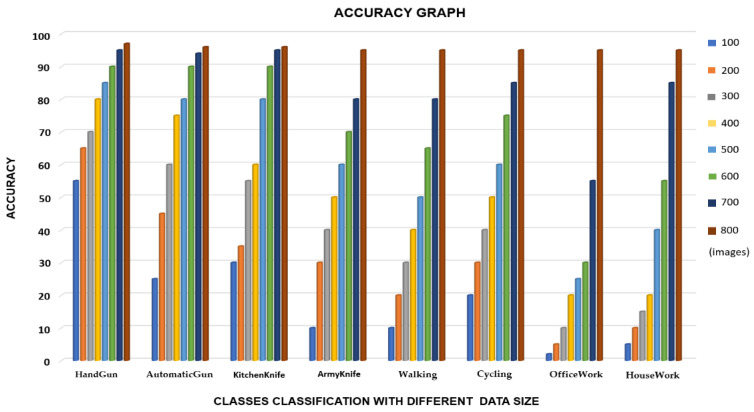
The accuracy of each subclass with respect to the trained batches of images. Colors representing each aspect are shown in the legend.

We used TensorFlow [37], which is a platform for training the neural network, and also Keras API (as a library for different transformations). As a front end, python programming was used, and training was conducted using Nvidia Geforce RTX 2060. For testing a model in real-time, a Logitech C920 Pro HD webcam was used. We also used the raspberry pi 4 to evaluate the feasibility of the MSD-CNN model on resource-constrained devices. For the used raspberry pi cameras, the camera feed was extracted from multiple slave raspberry pi and got processed on the master raspberry pi for abnormal object detection in sequence. After data augmentation on the dataset denoted as *M* was split into three parts, *Mtrain* set was used for training the algorithm, *Mdev* set was used for finetuning and for evaluating the variance and bias, and *Mtest* was used for checking the trained model’s precision. First, we used a smaller batch (100–200) of images for training. For this, the accuracy ranged from 20 to 30%. We increased the dataset batch size slowly after reaching a size of 800 images with 4000 iterations, and the accuracy achieved ranged from 90 to 99%, as shown in Figure 6 (the accuracy of each subclass with respect to the trained batches of images).

### 4.1. Materials

**Figure 7:**
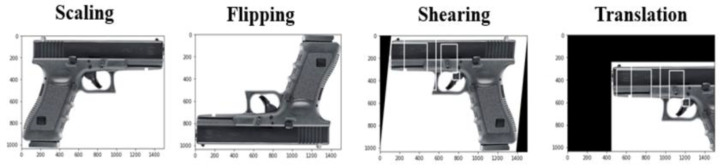
Data augmentation technique for dataset.

In this section, we describe in how the data augmentation technique, as shown in Figure 7, significantly increases training and testing datasets for detection.

#### 4.1.1. Dataset for Training

The dataset for the abnormal class and normal class is extracted from the ImageNet dataset and IMFDB. ImageNet dataset comprises 15 million labeled images that belong to 220,000 categories. A dataset comprises 58,000 different view images of guns. Thus, it increases the detection accuracy and robustness of the MSD-CNN model data augmentation method used on a dataset. Similarly, after augmentation on IMFDB images, we obtained 75,000 gun images. From the total obtained dataset, 75% of the data was used for training, 15% of the data was assigned to training validation, and the remaining 10% was used for testing.

- Flipping: Flipping an image is moving the image in a mirror-reversal horizontally or vertically;
- Shearing: Shearing an image is shifting part of an image;
- Scaling: Image scaling is the resizing of images.

With data augmentation, the dataset increased to 315,000 images for guns and the same for knives; the dataset increased to 175,000 images from 25,000 images. The images were extracted from the handgun, rifle, kitchen knife, army knife, cycling, walking, office work, and housework classes of the ImageNet dataset. Thus, the obtained dataset was an extensive custom dataset used for training.

Most of the images selected in the corresponding dataset for training comprise different viewpoints’ variation, illumination, deformation, occlusion, and interclass variation of images.

#### 4.1.2. Dataset for Testing

For guns and knives, testing sets were generated from existing datasets with a total of almost 48,000 images:

- The Open Image Dataset V4 comprised 55,000 handgun images [11] and 26,700 knives images;
- The small test set of Olmos [36] comprised 608 images.

### 4.2. Comparison of Qualitative Results

Evaluation and comparison of the existing studies with the proposed methodology are mentioned in Table 2; the parameters used for evaluating are the type of deployment [38] and off-time [15,16,18], which means the model used static data stored on the hard drive.

Real time [20,23,31] implies that the model is used on runtime where the data is a live camera video feed. We must also consider the detection type: different types of guns and knives (i.e., the model can detect and classify different types of guns and knives), handguns [15,16] (model can only detect handguns), knives [18,32] (model can only detect knife). Notably, until now, there has been no study on efficiently detecting and classifying different types of guns and knives; the neural network could not differentiate between different types of guns and knives with similar characteristics.

Most models lack robustness because they are trained on a very small dataset, ranging from 50 to 2400 images only. Considering camera view, most detection algorithms work properly on a single view camera [32] (use of a single camera with a single-object view); existing studies’ algorithms do not work on multiview cameras (using multiple cameras with multiple-object view) [32]. The proposed MSD-CNN model could achieve 90.7% precision with respect to parameters such as different types of guns and knives, real-time deployment, and multiview cameras.

### 4.3. Quantitative Results Analysis

We used a confusion matrix, which is a table for describing the performance of the classification model. Table 3 shows the result obtained by the classification model on the test set. The table determines the number of true positives (TP), the number of false positives (FP), the number of false negatives (FN), and the number of true negatives (TN). For the evaluation and comparison, the metric parameters used are precision, recall, and F1 measure [23], which can be defined as follows:

$$\mathrm{precision}=\frac{\mathrm{True}\;\mathrm{Positives}}{\mathrm{True}\;\mathrm{Positives}\;+\;\mathrm{False}\;\mathrm{Positives}}$$

$$\mathrm{recall}=\frac{\mathrm{True}\;\mathrm{Positives}}{\mathrm{True}\;\mathrm{Positives}+\mathrm{False}\;\mathrm{Negative}\;}$$

and

$$F1\;\mathrm{measure}=2\times \frac{\mathrm{Precision}\;\times \;\mathrm{recall}}{\mathrm{precision}+\;\mathrm{recall}}$$

Additionally, mean average precision (mAP) was also calculated for the test set. The mAP is an average precision over multiple intersections over the union. The obtained mAP is 97.50%. The result obtained on the Olmos [35] benchmark dataset is shown in Table 4. Notably, the model was not trained on the OpenImage Net V4 and Olmos dataset, and still, the model could detect and classify the guns and knives efficiently and effectively, with accuracy ranging from 90 to 98%. When the model was tested on this dataset, only the classes of data for handguns, knives, and walking were used. The OpenImage Net V4 dataset knife class mainly comprised kitchen knives, with the sharp edges of the knives defined in the image, which is also the reason for good accuracy. However, in the Olmos dataset, the occluded image was also detected accurately. Maximum FP during detection was observed in army knife occluded images with noise.

**Figure 8:**
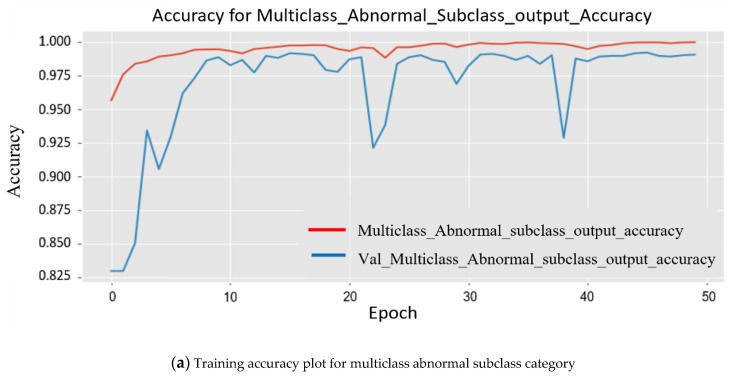

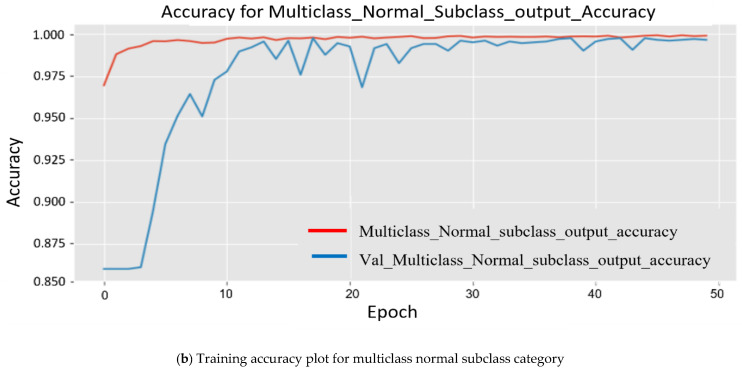
Two separate experimental plots show the accuracy in order to analyze the training.

**Figure 9:**
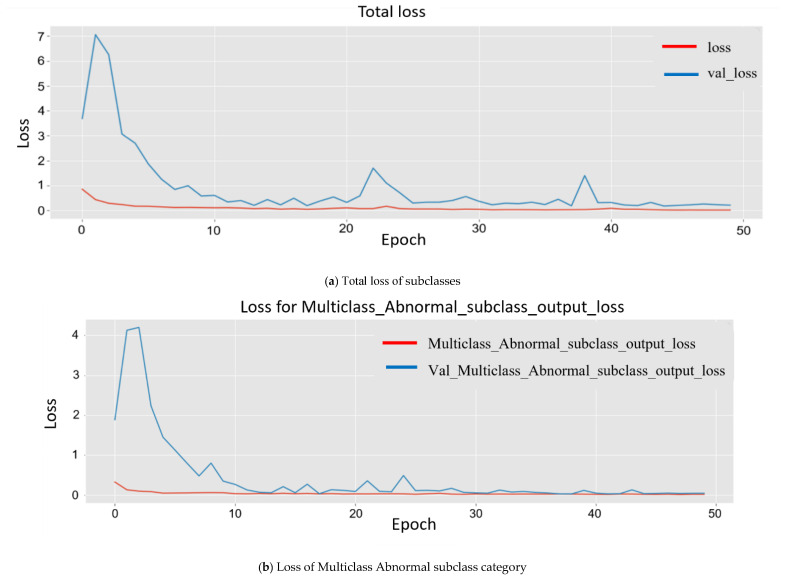

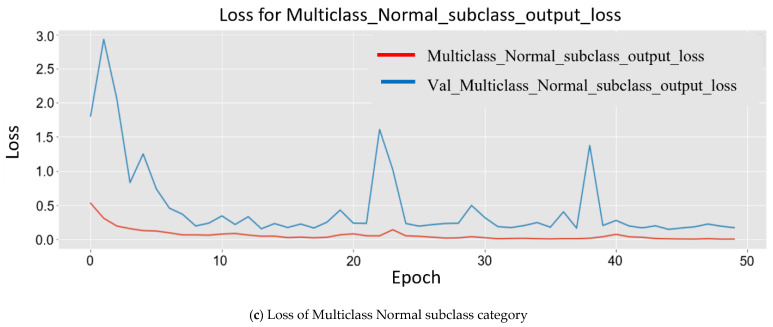
Experimental Training losses of multiclass subclass output classification plotted using matplotlib. They are plotted differently for analysis.

Since the model was trained on a larger dataset, while training the model, the results obtained for each major subclass are as follows. First, for the abnormal subclass, training set (*Mtrain*) accuracy was 98.90%, and 96.20% accuracy was obtained for the testing set (*Mtest*). Second, for the normal subclass, the training set (*Mtrain*) accuracy obtained was 98.60%, and 95.20% accuracy was obtained for the testing set (*Mtest*). The accuracy obtained was 96.20%, and 95.20% accuracy was obtained for the testing set (*Mtest*). The plot in Figure 8 shows multiple accuracies for the abnormal subclass and normal subclass. In Figure 9, the plots are given for multiple losses for both abnormal and normal subclasses. It also contains the total loss during training.

### 4.4. Detection Time Per Interval

**Figure 10:**
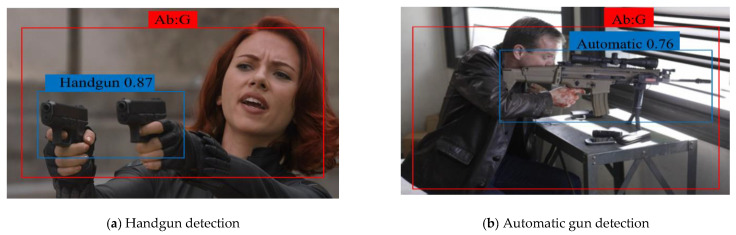

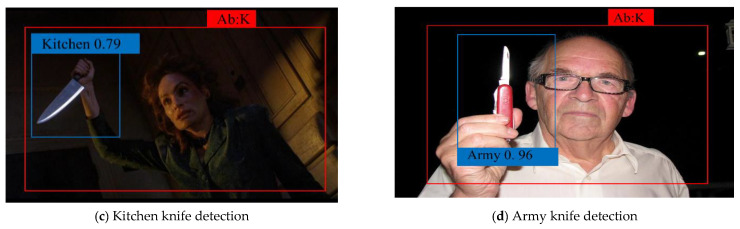
The accuracy of each subclass with respect to the trained batches of images. The red rectangle box determines an abnormal frame, and the blue rectangle box defines the detected subclass.

**Figure 11:**
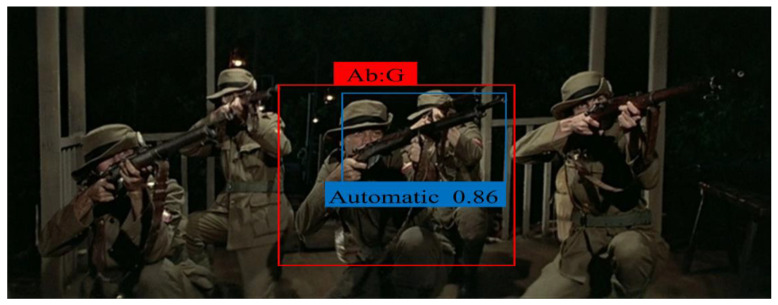
Depiction of a false negative, where there are three automatic guns in the background.

We propose a new parameter, DTpI, to evaluate the model in a real-time environment or a video. DTpI determines how accurately a model can detect the normal and abnormal subclass in the live video feed. DTpI can be denoted as i, and it indicates the TP frame that is being detected. For analysis, we used a publicly available YouTube video containing 20 frames of guns. In the video, the guns are clearly visible to a viewer. The MSD-CNN successfully detected i = = 18 frames, with an average time of DTpI = = 0.3 s. We intentionally tested the model on challenging videos, which contain occlusion and illumination. We tested the model on live camera feed and videos, and the model showed good performance in different scenarios. Figure 10 illustrates the detection accuracy of the abnormal subclass in the test videos. The detection result obtained on test videos shows two rectangle boxes. The red rectangle box determines an abnormal frame and its subclass gun, and the blue rectangle box defines a handgun subclass. The detection confidence score is 85% for Figure 10a. Similarly, other images depict the bounding box and its confidence score for the automatic gun and knife subclass. The false negative representation shows that the detected frames are low in brightness and contrast (as depicted in Figure 11). Specifically, they arise when the image quality is unclear or when the automatic gun is moved very fast in the background pixels. We can analyze that the accuracy of detecting smaller objects depends on the quality of the frame sequence.

Table 5 shows which images the model was trained on, which images the model was tested on, and the confidence score generated for each output image. For the first, the confidence score generated for detecting a handgun gun is 98%, and for the other remaining subclasses, the score is in the range of 0.1–18%, respectively. Thus, we can say that the model has perfectly detected the handgun. Similarly, for automatic/semi-automatic rifle output images, the score is 96.5; for kitchen knife output images, the score is 98.54%; for army knife output image, the score is 96.01%; and for all tested images, the score was in the range of 95–98.90% for abnormal subclasses. For the normal subclass, the confidence scores for detection are as follows: for walking output images, the score is 96.01%; for cycling output images, the score is 95.90%; for office output images, the score is 95.32%; and for work output images, the score is 95.20%. For the normal subclass, the confidence was in the range of 94–97%. Thus, most images were detected with a satisfactory score. A satisfactory score is where the model is confident in the detection and is greater than or equal to 50%. 

To evaluate the feasibility and efficiency of the proposed model, we compared the results with state-of-the-art networks such as YOLO [25] (defined as R1), SSD [39] (R2), RFCN [24] (R3), R-CNN [31] (R4), and FRCNN [23] (R5), which were tested on the customized dataset. For object detection, the R1 and R2 methods use regression techniques. R1 was fast while detecting the object, but it performed poorly in detecting tiny objects. In comparison with R1, R2 showed a minute difference in detecting the desired object. The techniques of R3, R4, and R5 showed better results than R1 and R2. It employs a sensitive position score map for classification; a major disadvantage of this technique is the computational cost, as it uses two-stage detectors. A comparison of results for each subclass is shown in Table 6.

As the study’s primary goal was to create a lightweight abnormal object detection model that can detect smaller objects such as guns and knives, we evaluated the proposed model on different platforms such as GPU, CPU, and on resource-constrained devices such as the raspberry Pi 4 model. To gain insights on different platforms, we compared the study with lightweight networks such as MobileNet [39] (defined as T1), and Tiny-YOLO [40] (T2). The rate at which the object is predicted can be determined by the inference and loading, which taken together represent the computational cost. The T2 method performed better than T1 and MSD-CNN when loading was considered. In contrast, MSD-CNN = 2.10 s performed slightly better than the T1 = 2.31 s and T2 = 3.0 s methods when inference was considered. The DTpI method was employed to check the inference in multiview cameras at the same time. We tested the MSD-CNN model on raspberry pi 4 for object detection in multiple cameras feeds, and the model outperformed the existing algorithm. A comparison of computational costs for different platforms is shown in Table 7.

In summary, we evaluated the model on different platforms for model effectiveness and efficiency and compared the result with different algorithms on a custom dataset for insights. The model exhibits good performance on different datasets.

## 5. Discussion

According to our results, a deep neural network can achieve a good result on a challenging dataset. Notably, the performance of the network degrades when a single convolution layer is removed. For clarity, suppose we are removing a midsection convolu’.tion layer; a drop of 4% is seen in the accuracy. So, for an accurate result, neural network depth is required, thus ensuring that the network is large enough to achieve the appropriate conclusion. For comparison, we revisited most of the detection studies, and we used standard methods such as precision, recall, F1 measure, and mAP on the benchmark dataset to evaluate the proposed methodology. Also, we proposed a new evaluation matrix (DTpI) for better understanding. We also distinguished that a consequential drop in accuracy exists when the dataset for training is too small, as the detected object would be sensitive and small. Besides this, a major disadvantage is that the model only works well on a very highly configured HD camera video feed. In such scenarios, the accuracy of detection was more comparable to the low-quality video feed/low-quality images. The detection was done on high-quality video; as the model is lightweight, less processing power is required.

The use of GPU and threading helps to achieve significantly better results compared with other algorithms. DTpI is employed to understand the inference of the detected object per camera. The model runs exceptionally well on resource-constrained devices for detecting the object in real-time for multiview cameras. Compared to FRCNN, MSD-CNN showed slightly better performance while detecting abnormal objects in multiview cameras for resource-constrained devices. More abnormal objects can be classified into different subclasses for detection. More extensive training can increase the robustness of the network. The detection works appropriately in a static camera view. This study can be extended to continuously moving cameras, which change in view direction. Sometimes the video frames are too dense or too overcrowded, and most views overlap in these scenarios, thereby making it difficult to detect.

## 6. Conclusions

Many criminal activities that occur in public areas involve the usage of a gun or knife. Video surveillance can be used for earlier detection of such events. In this study, we discussed the different methods used for detecting knives and guns, involving various parameters. We proposed an MSD-CNN model and tested it on benchmark datasets for detecting abnormal frames (dangerous events such as carrying guns or knives) and normal frames (events such as walking or officework). An essential advantage of the MSD-CNN is that it takes full account of tiny features in the images while training, thus detecting smaller objects more efficiently. Furthermore, as the MSD-CNN is a lightweight model, multiple model instances can be parallel executed on low-powered computational devices. In the proposed model, we considered two primary tasks: classification and automated detection of abnormal frames. For training the model, we significantly increased the existing datasets to make the model robust. Furthermore, the proposed model showed outstanding results in real-time scenarios. In addition, we evaluated the model on different computing platforms to check the feasibility of the model. A major disadvantage of the model is that it can detect only a specific set of objects on which it is trained.

Future work includes improving our method further, such as installing the model directly on the edge devices for computation. The future scope will be expanding this study to cloud and edge devices to distribute the computation power and make the model more robust for real-time scenarios. We plan to extend this algorithm to work on video synopsis to develop robust CCTV surveillance solutions based on testing the model on different sets of cameras, infrared cameras, low-resolution cameras, and continuously moving cameras. We are also obliged to include more sets of abnormal subclasses (e.g., hockey sticks, cylinders, and explosive materials) for detection.

## Figures and Tables

**Table 1:** Units for notation.

Symbol	Quantity
ⴖH	height
ⴖw	width
ⴖC	number of channels
M	training examples
Mtrain	number of inputs for training
Mtest	number of test images
W	weights
b	bias
X/a	input
Y	output (predicated value)

**Table 2:** Evaluation and comparison of different types of deep learning and classical non-deep learning algorithms for detecting guns and knives.

		Detection Type			Camera		Algorithm		Dataset		Evaluation Strategy			
	Deployment	Different Types of Gun & Knife	Handgun	Knife	Single View Camera	Multiview Camera	Non-Deep Learning	Deep Learning	Training	Testing	Max mAP	Precision	Recall	F1 Measure
Tiwari and Verma [15]	Off time	-	✔	-	✔	-	color-based segmentation and Harris interest point detector	-	CD	S	84.26	-	-	-
Glowacz et al. [18]	Off time	-	-	✔	✔	-	Harris Corner Detector	-	CD	S	92.5	-	-	-
Pratihar and Yadav [16]	Off time	-	✔	-	✔	-	Image Processing and the use of infrared rays	-	CD	S	-	-	-	-
Roberto Olmos [31]	Real time	-	✔	-	✔	-	-	Faster R-CNN	ID	S	84	-	96	91
Justin and Sydney [20]	Real time	-	✔	-	✔	-	-	Overfeat	ID	S	89	-	-	-
Verma and Dhillon [23]	Real time	-	✔	-	✔	-	-	Faster R-CNN	ID	S	93	-	-	-
Nakib et al. [32]	Off time	-	✔	✔	✔	-	-	CNN- Customized layer	ID	S	90	-	-	-
Kundergoski et al. [24]	Off time	-	✔	-	✔	-	-	RCNN, R-FCN, YOLOv2	XD	S	97	-	-	-
Zhang et al. [25]	Off time	-	✔	-	✔	-	-	RCNN, R-FCN, YOLOv2	HD	S	69	-	-	-
Bhatti et al. [30]	Real time	-	✔	-	✔	-		VGG16,YOLOv4	CD	CD	91	93	88	91
**The present work**	**Real, off time**	✔	✔	✔	✔	✔	**-**	**CNN-Customized layer**	**ID**	**S,D**	**97**	**90.7**	**86.3**	**95.7**

✔—Yes, CD—Customized Dataset, ID—ImageNet Datasets, HD—Human Datasets, XD—X-Ray Image Dataset, S—Same, D—Different (Open Image Dataset). Here, ‘same’ means a similar dataset is used for training and testing the model. The proposed model is trained on Image Dataset and is tested on Image Dataset and Open Image Dataset. The above result is based on the experimental evaluation of models with their corresponding data. All the parameters in the given table are explained in the qualitative result comparisons subsection.

**Table 3:** The results were obtained by classification model on the benchmark test set (*Mtest*).

Dataset	TP	FP	FN	TN	Precision	Recall	F1 Measure
ImageNet, IMFDB, and OpenImage Net V4	2305	61	40	394	90.7%	86.33%	95.7%

TP—True Positive, FP—False Positive, FN—False Negative, and TN—True Negative.

**Table 4:** Results obtained by classification model on Olmos test set.

Dataset	TP	FP	FN	TN	Precision	Recall	F1 Measure
Olmos	473	32	25	78	93.4%	94.66%	93%

**Table 5:** The confidence score for each subclass.

	Training Set	Testing Set	Result (%)	
Abnormal	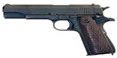	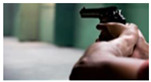	Ab:G: **handgun: 98** AbK: kitchen: 0.3 No:A: walking: 0.7 No:W: office: 0.1	Ab:G: automatic: 18 Ab:K: army: 0.6 No:A: cycling: 0.22 No:W: house: 0.07
	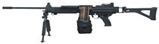	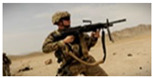	Ab:G: handgun: 10 Ab:K: kitchen: 0.5 No:A: walking: 0.4 No:W: office: 0.2	Ab:G: **automatic: 96.5** Ab:K: army: 0.3 No:A: cycling: 0.10 No:W: house: 1.0
	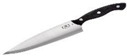	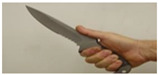	Ab:G: handgun: 6.2 Ab:K: **kitchen: 98.54** No:A: walking: 1.7 No:W: office: 0.15	Ab:G: automatic: 5.0 Ab:K: army: 30.5 No:A: cycling: 0.6 No:W: house: 0.1
	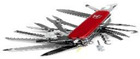	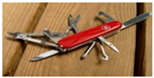	Ab:G: handgun: 0.3 Ab: K: kitchen: 11.1 No:A: walking: 0.32 No:W: office: 0.005	Ab:G: automatic: 0.10 Ab:K: **army: 96.01** No:A: cycling: 0.03 No:W: house: 0.02
Normal	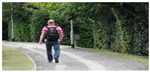	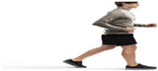	Ab:G: handgun: 0.009 Ab:K: kitchen: 0.1 No:A: **walking: 96.01** No:W: office: 0.57	Ab:G: automatic: 2.4 Ab:K: army: 0.65 No:A: cycling: 0.36 No:W: house: 2.7
	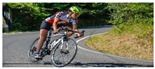	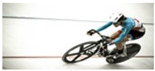	Ab:G: handgun: 0.26 Ab:K: kitchen: 0.21 No:A: walking: 0.63 No:W: office: 0.13	Ab:G: automatic: 0.01 Ab:K: army: 0.45 No:A: **cycling: 95.90** No:W: house: 0.35
	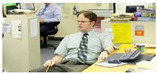	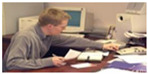	Ab:G: handgun: 0.18 Ab:K: kitchen: 0.17 No:A: walking: 0.78 No:W: **office: 95.32**	Ab:G: automatic: 0.032 Ab:K: army: 0.444 No:A: cycling: 0.006 No:W: house: 8.00
	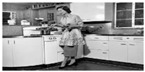	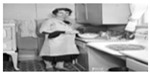	Ab:G: handgun: 0.54 Ab:K: kitchen: 0.16 No:A: walking: 0.01 No:W: office: 6.7	Ab:G: automatic: 0.78 Ab:K: army: 0.09 No:A: cycling: 0.13 No:W: **house: 95.20**

The confidence score during detection. Ab means abnormal subclass, No means normal subclass, G means gun subclass of abnormal class, K means knife subclass of abnormal class, A means activaty subclass of normal class. W means work subclass of normal class. For gun and knife class, there are each two subclasses each: handgun, automatic/semi-automatic rifles, kitchen knives, and army knives. Similarly, for activity and work class, there are two subclasses each: walking, cycling, office work, and housework.

**Table 6:** Detection average precision for each subclass with different algorithms.

		YOLO [R1]	SSD [R2]	RFCN [R3]	RCNN [R4]	FRCNN [R5]	MSD-CNN
Abnormal Frame	handgun	67.45	69.21	68.18	67.32	73.06	91.75
	automatic gun	75.32	70.54	74.21	72.13	78.54	94.71
	kitchen knife	55.45	53.08	58.71	60.36	71.44	89.23
	army knife	34.84	45.05	46.47	45.53	60.18	83.24
Normal Frame	walking	80.21	79.25	81.10	80.14	88.47	90.11
	cycling	81.54	84.23	86.98	85.74	89.36	89.47
	officework	78.25	77.55	85.68	88.22	89.21	91.75
	housework	82.14	79.11	87.36	89.56	90.12	90.46
	mAP	70.01	69.5	73.58	73.57	80.47	90.02

**Table 7:** The computational cost of detecting models on different computing devices.

	Mobile Net [T1]		Tiny-YOLO [T2]		MSD-CNN	
	Loading	Inference	Loading	Inference	Loading	Inference
Nvidia GeForce RTX 2060 (GPU)	0.0083	0.0031	0.0015	0.0033	0.0057	0.0019
PC-CPU	0.5	0.19	0.091	0.2	0.46	0.15
Raspberry Pi 4	2.97	2.31	0.6	3.0	1.86	2.10

Both loading and inference rate are defined in seconds.

## Data Availability

The data presented in this study are available on request from the corresponding author.

## Abbreviations

AAM	Active appearance model
CBS	Color-based segmentation
CNN	Convolutional neural network
COCO	Common object in context
DNN	Deep neural networks
GPU	Graphics processing unit
HCD	Harris corner detector
IMFDB	Internet movie firearm database
MSD-CNN	Multiclass subclass detection CNN
R-CNN	Region-based CNN
RELU	Rectified Linear Unit
SSD	Single shot multibox detector
UNODC	United Nations Office on Drugs and Crime
YOLO	You only look once
